# BARRIERS TO HEALTH CARE ACCESS AMONG UNDOCUMENTED IMMIGRANTS: PERSPECTIVE OF PALLIATIVE CARE HEALTH CARE PROVIDERS

**DOI:** 10.1093/geroni/igad104.2364

**Published:** 2023-12-21

**Authors:** Valeria Cardenas, Lindsey Hulsebus, Susan Enguidanos

**Affiliations:** University of Southern California, Los Angeles, California, United States; USC, Los Angeles, California, United States; University of Southern California, Los Angeles, California, United States

## Abstract

Undocumented immigrants face numerous barriers to obtaining healthcare services, largely due to fear of deportation and limited access to health care. The purpose of this study was to learn about challenges and facilitators palliative care healthcare providers experience in caring for seriously ill undocumented immigrants and the factors affecting access to care in this seriously ill population. From November 2022 to January 2023, we conducted semi-structured, individual telephone interviews with 11 palliative care healthcare providers from two inner city hospitals in California. Our interview protocol elicited providers’ experience caring for undocumented immigrants, including the patients’ stage of illness when providers encounter them, referrals made by the healthcare team, and gaps in care they have witnessed for these undocumented immigrants. Transcripts were analyzed using thematic analysis. Themes related to provider perceived patient care-seeking behaviors and challenges include complications of postponing care, financial burden, fear of deportation, transportation, health literacy, lack of family support, lack of awareness and misperceptions of palliative care. Themes related to provider care challenges include language barriers, longer care process, and cultural barriers. Facilitators and recommendations include awareness of care options and reassurance, importance of social and community resources, expanded palliative care awareness, knowledge, and access, and improved cultural competence. Our findings underscore the need for intervention studies that focus on alleviating identified barriers for undocumented immigrants. Future studies are needed to elicit the perspectives of seriously ill undocumented patients.

